# Effectiveness of Acupuncture in Treatment of Simple Obesity in Animal Models: A Systematic Review and Meta-Analysis

**DOI:** 10.1155/2019/5459326

**Published:** 2019-07-14

**Authors:** Xiao-lin Fan, Mei-ling Yu, Shu-Ping Fu, Yi Zhuang, Sheng-feng Lu

**Affiliations:** ^1^The No. 2 Clinical Medicine College, Nanjing University of Chinese Medicine, Nanjing 210023, China; ^2^Nanjing Vocational Health College, Nanjing 210023, China; ^3^Key Laboratory of Acupuncture and Medicine Research of Ministry of Education, Nanjing University of Chinese Medicine, Nanjing 210023, China

## Abstract

**Background:**

Simple obesity has become a global risk to health of human beings. Acupuncture, as one of traditional Chinese medicine therapies, has been widely used in obesity treatment in recent years. However, the individual heterogeneity which makes acupuncture's efficiency unstable leads to some controversy. So more evidence-based results are necessary to judge the effectiveness of acupuncture in treatment of simple obesity. Compared with clinical trials, animal experiments are controllable, and the underlying mechanism is more likely to be explored. Last but not the least, more and more experimental studies on acupuncture for animal obesity have been published. Therefore, we conducted the systematic review and meta-analysis to evaluate the effectiveness of acupuncture in treating simple obesity in animal experiments.

**Methods:**

Randomized Controlled Trials (RCTs) on acupuncture for simple obesity animal models were searched from six databases: PubMed, MEDLINE, CNKI, VIP, WanFang Date, and CMB from inception to February 2017 and updated on April 12, 2019. RevMan 5.3 software was used for meta-analysis. Treatment effects were summarized as relative risk (RR) and Standard mean difference (SMD) with 95% of confidence interval (CI).

**Results:**

A total of 108 trials involving 5731 rats were included. Meta-analysis showed that acupuncture had better effect on reducing weight (SMD -2.60, 95%CI: -2.93 to -2.26, p<0.00001) and Lee's index (SMD -2.62, 95%CI:-3.18 to -2.06, p<0.00001) compared with control group. However, the methodological quality of included studies was generally poor. Details of blinding were not reported in most studies. In spite of high heterogeneity being observed on the merged data, sensitivity analysis using the leave-one-out approach, subgroup analysis based on different acupuncture techniques, and rat strains and meta-regression all failed to find the sources of heterogeneity. The asymmetric funnel plot suggested publication bias. Besides, adverse events were not reported in any reports.

**Conclusions:**

Our review provided positive evidence of acupuncture for simple obesity. Unfortunately, none of the firm conclusions can be drawn due to methodological flaws, high heterogeneity, and publication bias. More high-quality trials are needed in future to get objective conclusions.

## 1. Introduction

Obesity, which is defined as being abnormal or excessive fat accumulation, accompanied with its complications, has been major risk for human health. A recent study that investigated the health effects of overweight and obesity in 195 countries over the past 25 years showed that the prevalence of obesity has doubled in more than 70 countries and has continuously increased in most other countries [1]. Simple obesity, the obesity which links to unhealthy lifestyles while having no other fundamental diseases, accounting for 95% of the obese, is the major risk factor for a number of chronic diseases, including diabetes, cardiovascular diseases, and cancer [1–3]. In terms of this global public health problem, Western medicine treatment mainly embraces weight loss drugs and surgery. The antiobesity drugs are considered to be taken for those patients whose BMI exceeds 30, or 27 accompanied with complications, but healthy diet, regular exercise, and behavioral intervention are still effective in curing obesity; for those patients whose BMI exceeds 40, or 35 accompanied with complications, surgical treatment is a suitable complementary therapy [4]. It is worth noticing that in antiobesity drugs only orlistat and sibutramine have been approved by the Food and Drug Administration (FDA) [5], and their therapeutic effectiveness and side effects still require long-term follow-up and clinical evaluation [6]. Although surgery is the best treatment from the viewpoint of significant and fast effect, few obese individuals opt for it because of high risk and high cost [7].

In view of Traditional Chinese Medicine (TCM), the primary pathogenesis of simple obesity includes internal stagnation of fluid dampness and meridian blockage with phlegm. The corresponding treatment principle is eliminating dampness and phlegm through invigorating the spleen [8]. As a form of alternative medicine and key component of TCM, acupuncture has been used widely throughout the world and its efficacy is recognized worldwide as shown in *«*Acupuncture: Review and Analysis of Reports on Controlled Clinical Trial*»* [9]. Actually, acupuncture has been widely used for treating simple obesity in clinic because of its benefits in various aspects such as lower cost, more remarkable curative ratio, persistent and stable effect, easy operation, and no regaining worries [10–12].

Modern medical research has shown that acupuncture is effective for simple obesity because of its effects on suppressing appetite, reducing food and energy intake, decreasing absorption of nutrients, and improving energy metabolism through regulating the neuroendocrine system, the digestive system, and the material metabolism [13–15]. There are plenty of clinical studies and researches to verify different acupuncture's effectiveness, including routine acupuncture [16], electroacupuncture (EA) [17], auricular point sticking therapy [18], cupping therapy [19], and acupoint catgut-embedding therapy [20]. Due to ethical considerations, clinical trials are unable to obtain detailed information of the acupuncture for obesity. Animal studies could not only limit external factors to get more accurate results but also investigate the underlying mechanism of the experiment. However, few methods can evaluate the clinical applicability of animal experimental studies. The systematic review of animal experiments, which revaluates animal trials through statistical approach to avoid unreasonable decisions is considered as an effective method to explore and improve the guiding value of animal experiments for clinical research [21]. Therefore, we conducted a systematic review and meta-analysis of the effectiveness of acupuncture for losing weight in animal models.

## 2. Materials and Methods

### 2.1. Eligibility Criteria

#### 2.1.1. Types of Studies Included

This systematic review collected randomized controlled trials (RCT) and limited language to Chinese and English.

#### 2.1.2. Types of Subjects

Animal models of simple obesity were included.

#### 2.1.3. Types of Intervention

Acupuncture was the main therapy without restriction on acupuncture types or treatment duration.

#### 2.1.4. Types of Outcomes

Weight and Lee's index (tested at the end of treatment) were chosen as the primary outcomes to evaluate the effectiveness of acupuncture. Weight and Lee's index are classic tests to reflect degree of animal obesity and have been widely applied to animals' experiments.

### 2.2. Search Strategy

The following six electronic databases were searched from inception to February 2017 and updated on April 12, 2019: PubMed, Medline, the China National Knowledge Infrastructure (CNKI), Chinese Science and Technology Periodical Database (VIP), WanFang data Information Site, and Chinese Biology Medicine Disc (CBMdisc). All searches were restricted on animal experiments but without limits to publications. The search terms utilized consisted of two groups with the medical subject headings: intervention (acupuncture and other related terms) and object (simple obesity and other related terms) in both Chinese and English databases.

### 2.3. Studies Selection

Only those studies which assessed the use of acupuncture for simple obesity in animal models were included in this review. Two authors (XLF and MLY) independently screened literatures from databases and listed the titles according to the above eligibility criteria. Two reviewers (XLF and MLY) independently carried out the next studies selection, discussed their differences, assessed the risk of bias, and extracted data of the selected articles. Any inconsistency in this process was discussed with the third person (SFL)

### 2.4. Data Extraction

Two authors (XLF and MLY) independently conducted data extraction and the controversy was resolved by discussion. The last data was compared from the following four aspects: (1) basic information, including the name of the first author, the year of publication, and model of simple obesity; (2) baseline characteristics of animal models, including the species, number, and weight; (3) aspects of treatment, including duration, frequency, course of treatment, and details of acupuncture; and (4) main outcomes (weight and Lee's index) and intergroup difference.

### 2.5. Quality Assessment

Two authors (XLF and MLY) used a ten-item scale to evaluate the methodological quality of the included studies. Ten items included peer-reviewed publication, control of temperature, exclusion of stress reaction, random allocation to treatment or control, blind method, sample size calculation, compliance with animal welfare regulations, statement of research support, detailed modeling method, and complete testing data. The study got one quality score as it fulfills each criterion of the item; the final score ranges from zero to ten. The higher the score, the higher the article quality. Any disagreement was discussed with the third author (SFL).

### 2.6. Statistical Analysis

RevMan 5.3 software was used for meta-analysis. Treatment effects were summarized as relative risk (RR) and Standard mean difference (SMD) with a 95% of confidence interval (CI). The Standard mean difference (SMD) was an evaluation of the combined effect sizes and P values below 0.05 were considered statistically significant. Heterogeneity was calculated with I-squared statistics and *χ*-squared distribution. Besides, we carried out subgroup analysis based on different acupuncture methods and different animal species. To explore the sources of heterogeneity, we did the subgroup meta-analyses and metaregression. The funnel plot and Egger's and Begg's Test were applied to detect the publication bias.

## 3. Results

### 3.1. Study Inclusion

**Figure 1:**
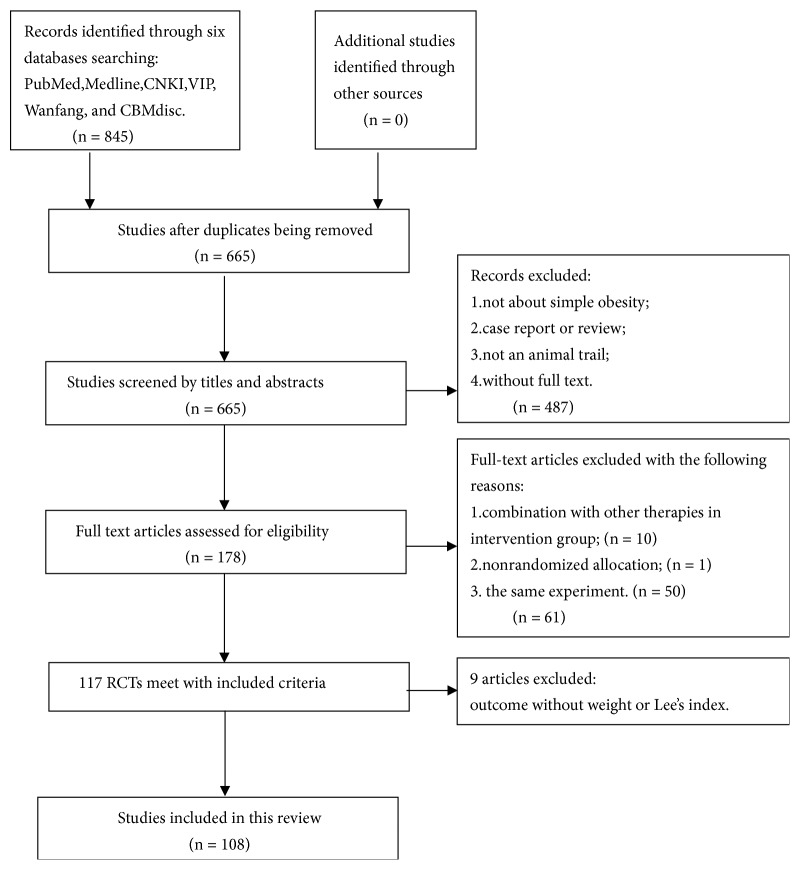
Flow diagram of the study selection process for this systematic review and meta-analysis.

We identified 845 potentially relevant articles before February, 2017, from the following six databases: PubMed, Medline, the China National Knowledge Infrastructure (CNKI), Chinese Science and Technology Periodical Database (VIP), WanFang data Information Site, and Chinese Biology Medicine Disc (CBMdisc). After removal of duplicates, there were 665 records remaining. Based on review of titles and abstracts, we ruled 487 papers out because of at least one of the following reasons: (1) not being about simple obesity, (2) case report or review, (3) not an animal trail, and (4) being unable to get full text. After reading all the remaining 178 articles through, 61 studies were excluded for the following considerations and issues: (1) combination with other therapies in control group, (2) nonrandomized allocation, and (3) republication for the same experiment. Among the included 117 studies, 9 studies were ruled out because their main outcome measures consisted of neither weight nor Lee's index. At last, 108 studies were included. The flow diagram of the study selection process is shown in Figure 1.

### 3.2. Study Characteristics

101 studies were written in Chinese and 7 in English. The 7 English studies were all published in international journals and the other 101 Chinese studies all in Chinese Journals. The 108 included studies involve a total of 5731 animals (74 studies with SD rats, 25 studies with Wistar rats, 8 studies with mice, 1 study with guinea pigs, and 3 studies which did not mention specific types of rats). Of all included trials, 13 studies did not provide the specific data of weight. The weight of rats ranges from 40g to 120g in 63 studies and 130 to 250g in 22 studies. The mice in 7 experiments weighed from 9 to 24g. In terms of gender, the vast majority of trials selected male rats; only 3 trials chose female rats. 12 studies used both male and female rats and 9 studies did not mention the sex. Most of trials (104) induced simple obesity by feeding with high fat diet, 2 trials by sodium glutamate, and 2 trials adopted simple obesity rats directly. Most of the rats, having eliminated stress reaction, were randomly assigned to acupuncture group or control group. Of these included RCTs, 77 studies used electroacupuncture, 16 studies chose handle acupuncture, and 11 studies adopted catgut implantation while 4 studies used auricular acupuncture. No treatment was given to the control group. A total of 56 studies used both weight and Lee's index as main outcome measures. Meanwhile, 48 studies only employed weight and 4 studies only used Lee's index as the outcome measure. Of the four studies with auricular acupuncture, except two studies without specific points, the other two studies chose the following areas on the auricle: Gastric, Small Intestine, Stomach, and Hunger Point. Within all the remaining 104 studies, besides 6 studies which did not give concrete points, we sorted out about 17 acupoints used in experiments: Zusanli (ST 36), Neiting (ST44), Zhongwan (CV 12), Tianshu (ST 25), Sanyinjiao (SP 6), Guanyuan (CV 4), Fenglong (ST 40), Yinlingquan (SP 9), Neiguan (PC 6), Quchi (LI 11), Daheng (SP 15), Zulinqi (GB 41), Weishu (BL 21), Sibai (ST 2), Liangmen (ST 21), Pishu (BL 20), and Guilai (ST 29). Then, we generate a list to calculate the times and frequency of acupoints and meridians used. The leading acupoints Zusanli (ST 36), Zhongwan (CV 12), Neiting (ST 44), Tianshu (ST 25), Sanyinjiao (SP 6), Guanyuan (CV 4), Fenglong (ST 40), and Yinlingquan (SP 9) adopted times were 88, 36, 34, 33, 26, 41, 12, and 6, respectively. Correspondingly, the most common used meridian was the Stomach Meridian of Foot-yangming, followed by Conception Vessel and the Spleen Meridian of Foot-Taiyin.

Based on different acupuncture techniques, the frequency and course of treatment were quite different. For catgut implantation therapy, the rats were treated once around every 7~15 days for almost 2~4 cycles. The course of treatment in electroacupuncture, handle acupuncture, and auricular acupuncture therapy were similar, 10 to 30 minutes of retaining needle basically, almost once or twice a day, lasting 8 to 56 days.

Of all the 77 studies that adopted electroacupuncture, only 2 studies ignored the detailed operating parameters. 36 trials, more than half of the studies, used continuous waves with frequency of 1-100 Hz. 14 studies used disperse-dense waves with frequency of 2-100Hz and 1 study used discontinuous wave with a frequency of 2Hz. The rest 25 studies provided the frequency of waves but referred no specific wave types. It could be found that continuous wave, 10Hz of frequency, was the most commonly used stimulus parameter of electroacupuncture for obesity. Even reinforcing reducing by twirling was the commonly adopted manipulating technique in manual acupuncture groups. The basic characteristics of the 108 studies are shown in Table 1.

### 3.3. Study Quality

The study quality checklist items score ranged from 3 to 8 out of total 10 points, which includes such items as peer-reviewed publication, control of temperature, random allocation to treatment or control, blinding, stress reaction, sample size calculation, detailed molding methods, complete outcome data, compliance with animal welfare regulations, and statement of potential conflict of interests or foundation items. Of the 108 included studies, one study got 3 points; twelve studies got 4 points; forty-two studies got 5 points; thirty-five studies got 6 points; sixteen studies got 7 points and one study got 8 points. Seventy-seven studies were rigorously peer-reviewed before publication. In fifty trials, temperature was controlled while one study did not describe the explicit temperature. Randomization was performed in all trials while only one study reported blinding. Two studies failed to rule out stress reaction. No trial reported sample size calculation. Ninety-nine studies described detailed methods of establishing the animal model including process and time consuming. One hundred and two studies gave the complete results and analysis of those experiments. Forty-two studies mentioned their foundation items (Table 2).

### 3.4. Effectiveness

**Figure 2:**
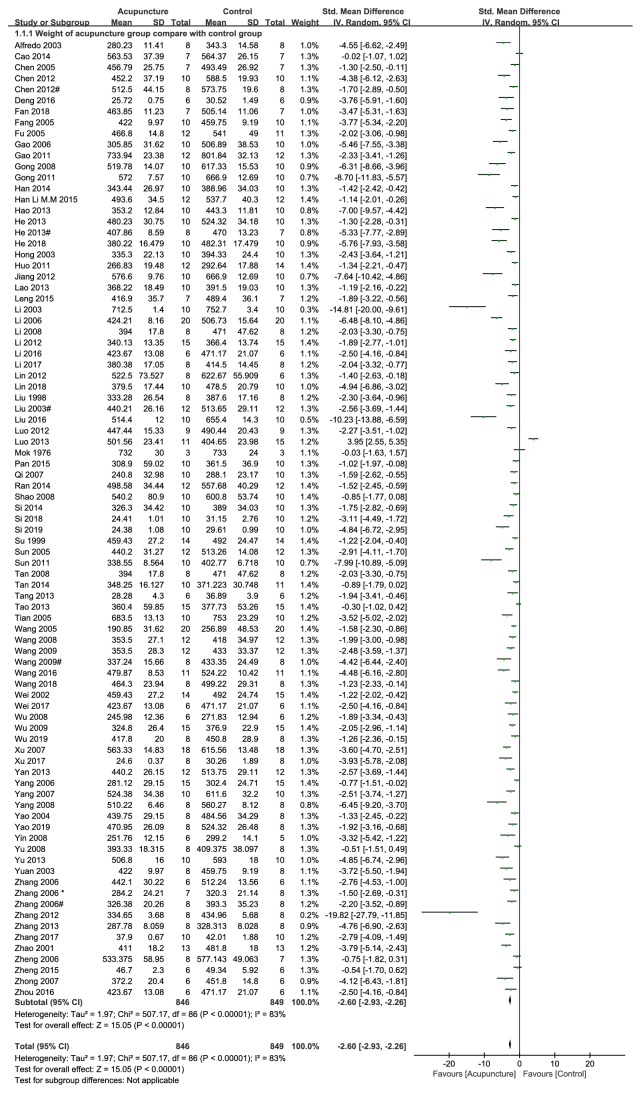
The forest plot of outcome measure ‘weight after intervention.'

Eighty-seven studies showed significant beneficial effects of acupuncture for losing weight compared with control group (n = 1695, SMD -2.60, 95%CI: -2.93~-2.26, p<0.00001; heterogeneity X^2^=507.17, p<0.00001, I^2^=83%, Figure 2); although the left seventeen studies also reported effects of acupuncture on losing weight in comparison with control group, they failed to be incorporated into meta-analysis because of lacking specific data to produce a data graph (p<0.05 or p<0.01).

**Figure 3:**
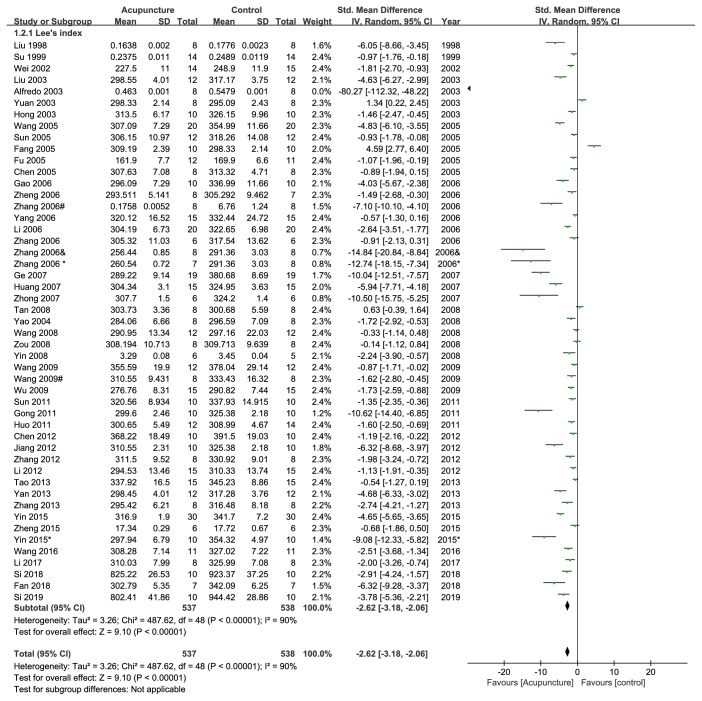
The forest plot of outcome measure ‘Lee's index after intervention.'

In sixty of included publications, Lee's index was reported after acupuncture, but ten of them have no statistical analysis due to short of concrete data (p<0.05 or p<0.01); the other forty-eight publications reported statistically significant effect of acupuncture on decreasing Lee's index compared with the control group (n =1075, SMD -2.62, 95%CI: -3.18~-2.06, p<0.00001; heterogeneity X^2^ = 487.62, p<0.00001, I^2^=90%, Figure 3).

However, large heterogeneity on the merged data was hard to ignore (heterogeneity of merged weight: I^2^=83%, p<0.00001; heterogeneity of merged Lee's index: I^2^=90%, p<0.00001).

### 3.5. Sensitivity Analysis

We made sensitivity analysis on the merged data, which did not decrease heterogeneity.

### 3.6. Subgroup Analysis

Since different acupuncture techniques and rat strains were employed by the included studies, we carried out subgroup analyses stratified by the two factors on weight of rats, respectively. A random effect was used for statistical analysis. Heterogeneity was still great in each group.

**Figure 4:**
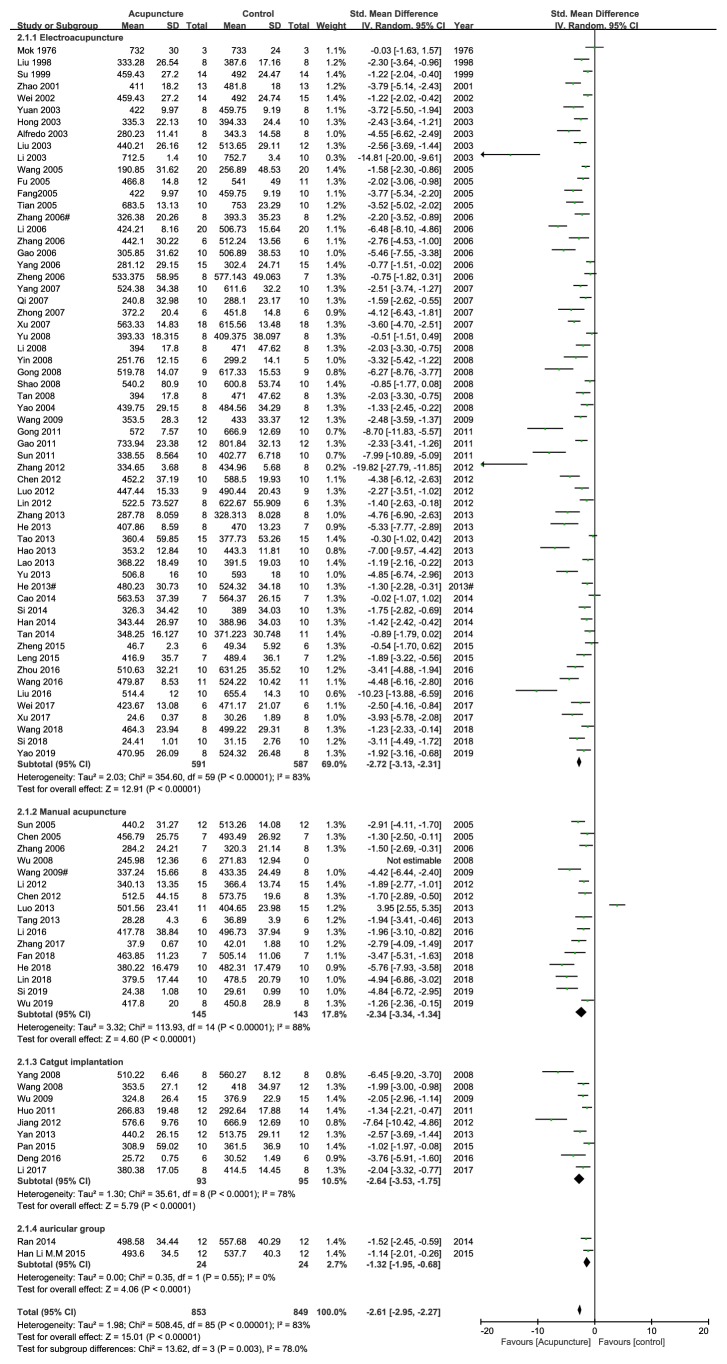
The forest plot of outcome measure ‘weight after intervention' in subgroup according to different acupuncture methods.

There are sixty articles compared electroacupuncture against other interventions, and the results showed high heterogeneity (heterogeneity X^2^=354.60, I^2^=83%, Figure 4). It also indicated that electroacupuncture was more effective than other interventions in losing weight (n =1178, SMD -2.72, 95%CI: -3.13 ~-2.31; Z=12.91, p<0.00001, Figure 4). Handle acupuncture group versus control group was tested in 16 studies. Great heterogeneity was found in this subgroup (heterogeneity X^2^=113.93, I^2^=88%, Figure 4). Handle acupuncture group showed more reduction in weight compared with control group (n = 288, SMD-2.34, 95%CI:-3.34 ~-1.34; Z=4.60, p<0.00001, Figure 4). Nine studies showed that catgut implantation tends to lose more weight than control group (heterogeneity X^2^=35.61, I^2^=78%; n=188, SMD-2.64, 95%CI:-3.53 ~-1.75; Z=5.79, p<0.00001, Figure 4). Two studies showed that auricular acupuncture tends to lose more weight than control group (heterogeneity X^2^=0.35, I^2^=0%; n=48, SMD-1.32, 95%CI:-1.95 ~-0.687; Z=4.06, p<0.0001, Figure 4).

**Figure 5:**
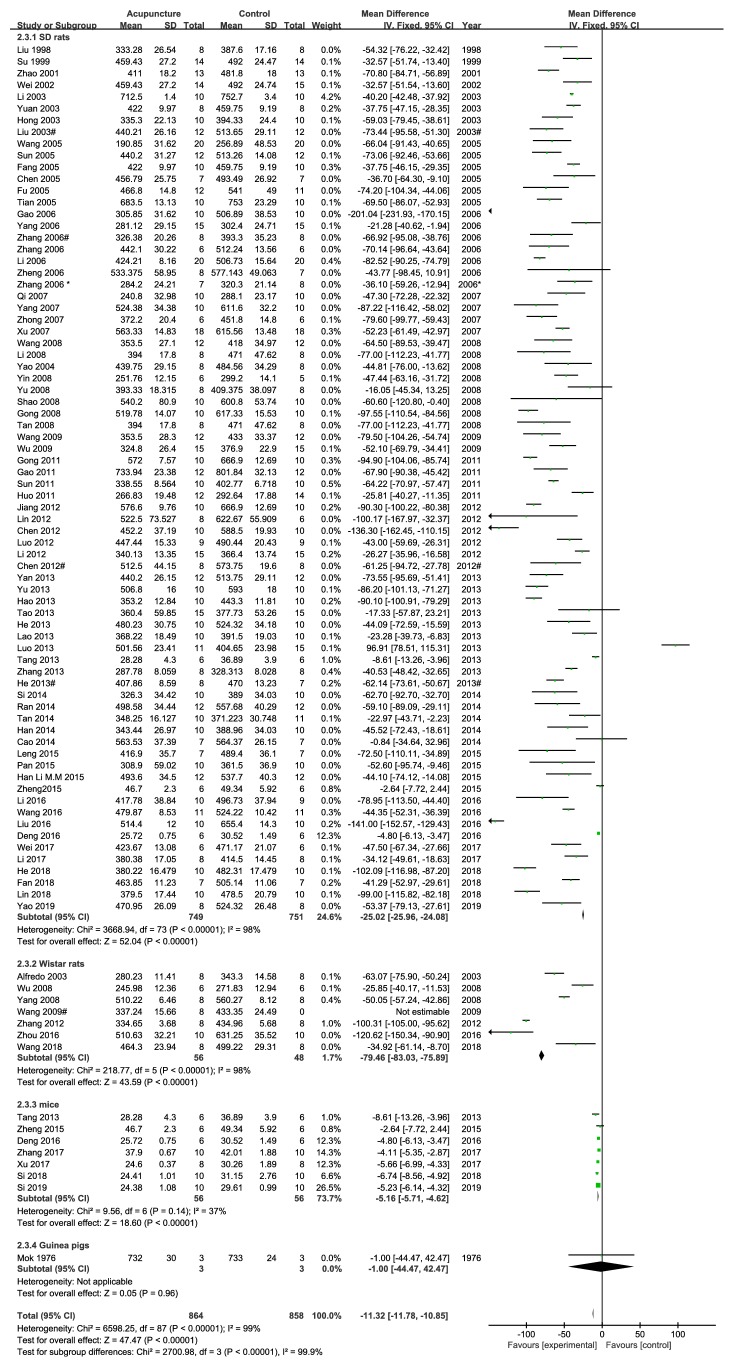
The forest plot of outcome measure ‘weight after intervention' in subgroup according to different species.

Based on different rat strains, the included studies were divided into four subgroups: SD rats, Wistar rats, mice, and guinea pigs. High heterogeneity was noted in both the subgroup of SD and Wistar rats but low in the mice subgroup (SD rats subgroup: I^2^=73%, p<0.00001; Wistar rats subgroup: I^2^=98%, p<0.00001; mice subgroup: I^2^=37%, p<0.00001, Figure 5).

### 3.7. Metaregression

**Figure 6:**
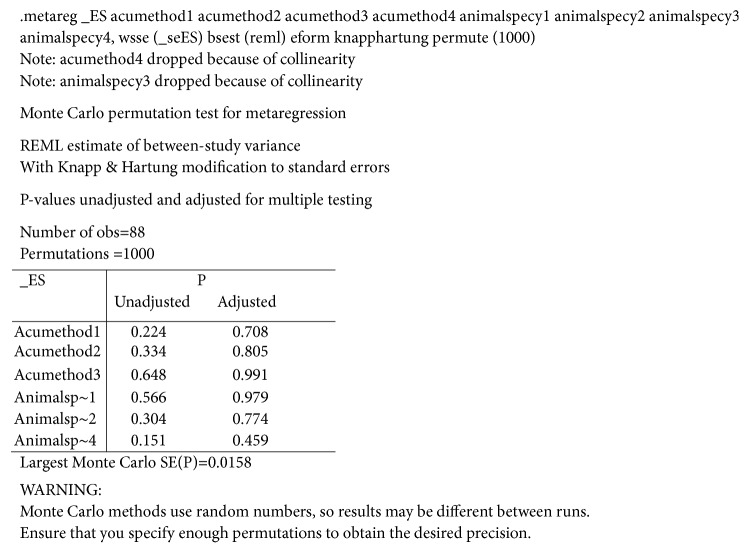
The results of the meta-regression employed acupuncture methods and rat strains as covariates.

The meta-analysis indicated that acupuncture group was superior to control group in losing weight and controlling Lee's index; unfortunately, significant heterogeneity was observed. As a result, we adopted further multifactor metaregression to explore the sources of heterogeneity by taking acupuncture methods (handle acupuncture, electroacupuncture, and catgut implantation) and rat strains (SD rats, Wistar rats, and mice) as covariates. However, the results of metaregression showed that acupuncture methods and rat strains were irrelevant to heterogeneity (Figure 6).

### 3.8. Assessment of Bias

**Figure 7:**
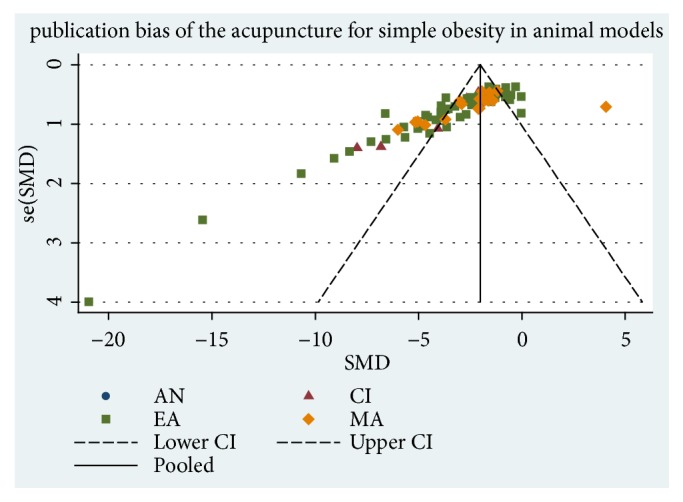
Funnel plot for publication bias analysis.

**Figure 8:**
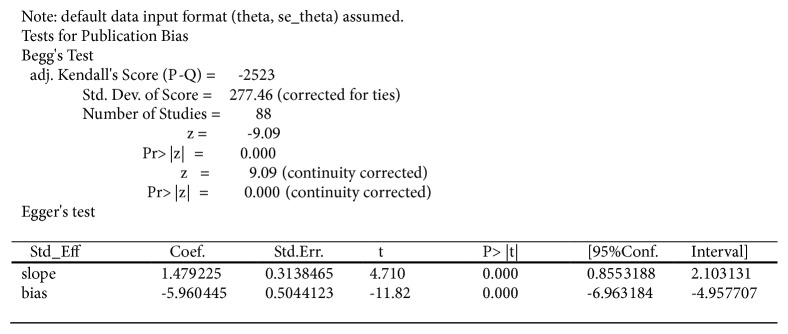
Egger's and Begg's Test for publication bias analysis.

We assessed publication bias with funnel plot and Egger's and Begg's Test. The asymmetry funnel plot suggested that there was publication bias (Figure 7). Quantitative evaluation by Begg's and Egger's Test showed that significant publication exists( Pr >|z|= 0.000, P >|t|= 0.000, Figure 8).

## 4. Discussion

### 4.1. Principal Findings

To our knowledge, this is the first systematic review and meta-analysis to assess the efficacy of acupuncture for animal model of simple obesity with weight and Lee's index as the main outcome measures. This meta-analysis indicated that acupuncture could have a certain effect on simple obesity, including losing weight and reducing Lee's index, and the conclusion is similar to previous clinical meta-analysis and systematic reviews [22–26].

### 4.2. Possible Therapeutic Mechanism of the TCM

“Dan Brook Heart Law,” one of the classical works of TCM in China, holds that obese people are more susceptible to phlegm dampness. The primary pathogenesis is internal stagnation of fluid dampness and meridian blockage by accumulated phlegm, which has close relationship with spleen, stomach, kidney, and large intestine. Therefore, eliminating dampness and phlegm through invigorating spleen is the basic principle of treatment [27]. The selected acupoints are almost all belonging to the Stomach, Spleen, and Conception Vessel (96.25%). It is just in line with the basic pathogenesis of turbid sputum, which has a key role in ensuring effectiveness by eliminating dampness and phlegm through invigorating spleen, harmonizing Yin and Yang, and dredging channel of Qi and Blood.

Zusanli (ST 36), the most frequently used he-sea point of Stomach Meridian of Foot-Yangming, can enhance the function of spleen and stomach, eliminate phlegm, and remove dampness. Lots of experimental results illustrated that acupuncturing of Zusanli (ST 36) can promote gastrointestinal wriggle, improve gastrointestinal motility, and regulate gastrointestinal function [28, 29]. Research [30] suggested that electroacupuncture on Zusanli (ST 36) or Neiting (ST 44) could suppress appetite and stave off hunger by stimulating beta-receptors; meanwhile, acupuncture has anticholinergic functions. Neiting (ST 44), as spring point of Stomach Meridian of Foot-Yangming, is good at clearing damp heat in the stomach and intestine specially. Zhongwan (CV 12) is a front-mu point of the Stomach, as well as an influential point of fu-organs and an intersecting point of the Small Intestine, Stomach, Triple Energize, and Conception Channel. Therefore, it can be used to treat all spleen and stomach diseases by regulating activities of qi, ascending lucidity, and descending turbidity. Acupuncture in Zhongwan point is valuable to regulate gastrointestinal function and gastric acid secretion because afferent neurons in CV12 and gastrointestinal tract overlap morphologically in the spinal ganglions of T7-L2 [31]. Tianshu (ST 25), located at the abdomen, is the most common place for body fat to accumulate. So acupuncturing in Tianshu has a better local stimulation effect for promoting abdominal fat to decompose [32, 33]. As front-mu point of the Large Intestine Meridian of Hand-yangming, Tianshu (ST 25) is beneficial for clearing away the pathogenic heat of the large intestine, removing stagnancy, regulating qi, and relaxing the bowels. Therefore, Tianshu (ST 25) is a preferred acupoint for simple obesity with Stomach-intestine Excessive Heat Type, whose symptoms include constipation, lack of body fluid, and feces dry knots. As Tianshu (ST 25) is just located on the body surface projection of gut, stimulation of acupuncture more easily passes into the intestine to adjust gastrointestinal motility, absorption, and secretion. For its biphasic regulation and excellent therapeutic effects for intestinal disease, Tianshu (ST 25) has been widely used clinically [34–36]. Sanyinjiao (SP 6), an intersecting point of the Liver, Spleen, and Kidney Meridians, is an important point to treat diseases of the three relevant organs by harmonizing liver, invigorating spleen and kidney, removing dampness, and clearing away turbidness. As a result, acupuncture of Sanyinjiao can provide better curative effect for obesity caused by retention of water, dampness, phlegm, and fluid caused by liver stagnation or deficiency of spleen and kidney. Research [37] has shown that acupuncture of Sanyinjiao can reduce the content of cholesterol in the blood by restraining its synthesis and absorption, accelerating its decomposition and excretion, and changing its distribution in the plasma and tissue. Guanyuan (CV 4), located on 3 inches below the navel and right at the pubic region where the root of human life is originated, is very effective in curing any injury detriment of Primordial Qi. Through reinforcing vitality and strengthening primordial qi, promoting blood circulation to resolve turbid-phlegm, Guanyuan is especially suitable for simple obesity resulting from the deficiency of Qi. Its curative effect is confirmed by many researches [38, 39].

### 4.3. Possible Mechanism from Modern Medicine Aspect

Combining the above analysis and numerous research results [12, 40, 41], the possible modern mechanism of acupuncture for simple obesity includes but is not limited to the following. Acupuncture can inhibit excitatory digestion in stomach and intestine and suppress appetite in obese patients. On the other hand, acupuncture can promote energy metabolism and increase energy expenditure to accelerate lipolysis. The included animal experiments in this paper also revealed the role of acupuncture in treating simple obesity. Acupuncture has optimal regulation on body's weight [42–46], lipid metabolism [47–50], blood fats [51–55], insulin [56–58], electrogastrogram (EGG) [27], and nucleus of hypothalamus. The possible central mechanisms of acupuncture for simple obesity are as follows: (1) neurons discharge regulation: acupuncture can reduce the excitation of lateral hypothalamic area (LHA) and increase electric activity frequency in ventromedial hypothalamus (VNH). So acupuncture could achieve weight loss through appetite suppressing and calories cutting [57, 59]; (2) modulation of the neurotransmitter: acupuncture can normalize hypothalamus ingestion central functions by adjusting content of monoamine neurotransmitters including catecholamines and 5-hydroxytryptamine (5-HT) [60, 61], cholecystokinin (CCK) and vasoactive intestinal peptide (VIP) [62], neuropeptide Y (NPY), and leptin (LP) [27, 54, 62–75]; (3) improving insulin resistance: acupuncture can increase the number and affinity of insulin receptor to improve insulin resistance (IR) status. So it is advantageous for reversing the metabolic disorders of glucose and lipids [56, 58, 76–82]. However, the conclusion of this meta-analysis still should be taken prudently due to the publication bias, significant heterogeneity, and other limitations.

### 4.4. The Value of Animal Studies and Systematic Review of the Effectiveness of Acupuncture for Obesity in Animal Model

It is well known that acupuncture is safe and effective for obese people to some extent. But controversy does exist for acupuncture's placebo effect. Revealing the mechanisms of acupuncture for obesity and making sure that the effect is not just placebo are significant for the development of acupuncture. To uncover these puzzles, it is necessary to objectively study the effects of acupuncture on fat and related tissues, cells, and metabolic signal pathways. As we know, it is unethical and impossible to get human's tissues and cells optionally. So, animal studies of acupuncture for obesity are of great value and cannot be replaced completely by clinical research. It should be noticed that all animal studies for potential mechanism of obesity treated by acupuncture depend on a precondition: acupuncture is effective for obese animals definitely. In general, applying the method of systematic evaluation to evaluate the experimental research of acupuncture is of great significance for improving the basic research level and quality of acupuncture. Therefore, we conduct this systematic review and meta-analysis to assess the effectiveness of acupuncture in treatment of simple obesity in animal models.

### 4.5. Limitations

Firstly, the vast majority of the included studies were published in Chinese; few studies were published in English. We did not include studies in other languages, which may cause certain degree of publication bias. Most of the included studies came from China because acupuncture as a key component of TCM is widely used in China but not so popular in other countries.

Secondly, no ‘negative' trial was included in our meta-analysis, which may induce potential publication bias. This might be because negative data is hard to publish compared with positive data. As a result, we may miss some negative data, which could lead to an overestimate of the effectiveness of acupuncture for simple obesity.

Thirdly, the methodological weakness of included trials also prevented drawing definitive conclusions as follows. (1) Randomization: in all the trials, we selected and adopted the principle of randomization, but the details of randomization procedures were often absent. That is why we could not give direct judgement. (2) Blinding: only one of the enrolled trials used blinding method, which may cause bias to some extent. (3) Low quality and small numbers of studies: low quality of the included articles and small number of studies with specific acupuncture methods (such as auricular acupuncture) were certain limitations to our analysis. (4) Lacking unified diagnostic criteria: to our knowledge, acupuncture is a key component of TCM, so it is best to apply acupuncture based on TCM syndrome. However, a considerable part of the included studies used “modern diagnosis” instead of “TCM syndrome” as the diagnostic criteria.

Besides, the substantial heterogeneity should be taken into consideration. Despite sensitivity analysis, subgroup analysis, and multifactor metaregression all performed, the factors that caused high heterogeneity have not been found yet. There are many reasons contributing to the generation of heterogeneity, such as the intramuscular needle insertion with different angles, depths and locations, different types of needles, different needling techniques of different operators, time of retaining needle, and scheduled time interval of acupuncture.

### 4.6. Implications for Future Trials

Further studies should recognize and overcome these referred limitations by the following aspects. Firstly, future studies should be encouraged to post negative data, side effects, and complications to reflect the effect of acupuncture objectively.

Also, rigorously designed RCTs and improved methodology are required. Randomization and blinding should be performed strictly. Future trials should attempt to expand sample size of trials with special acupuncture and adopt more unified TCM diagnostic criteria.

Moreover, a unified standard of acupuncture therapy should be taken seriously since details of acupuncture manipulation closely are related to the efficacy. Standardized acupuncture protocols, acupuncture techniques, treatment course, outcome measures, and even highly skilled acupuncturist are necessary.

## 5. Conclusion

Although our review provided positive evidence that acupuncture seems to be an effective therapy for simple obesity, it should be noted that the evidence justifies more future high-quality studies due to the possibility of publication bias and significant heterogeneity of the included studies.

## Figures and Tables

**Table 1:** Characteristics of eligible studies.

First author (year)	Species (sex)	No. of rats (intervention/control group)	Weight(g)	Model method (time)	The main points	Course of treatment	Intervention group	The main outcomes	Intergroup difference
Yan (2015)[27]	Wistar rats	(8/8)	180 ± 20	High-fat diet (8w)	ST36, CV12	20 minutes, once a day, 4 weeks	Electroacupuncture: continuous waves of 20Hz of frequency and current density of 1.5V, 4mA	1.Weight 2.Lee's index	1.P<0.05 2.p<0.05
Yin (2015)[83]	Rats	(30/30)	Not given	High-fat diet	SP6, ST36	10 minutes, once a day, 15 days	Electroacupuncture: continuous waves of 10Hz of frequency and current density of 1V	Lee's index	P<0.05
Yin (2015)[84]	SD rats(male)	(10/10)	180-230	High-fat diet (6w)	BL21, CV12	20 minutes, once a day, 3 weeks	Electroacupuncture: disperse-dense waves of 10-100Hz of frequency	Lee's index	P<0.01
Ran (2014)[47]	SD rats (male)	(10/10)	Not given	High-fat diet (8w)	Gastric and small intestine	30 minutes, 4 weeks	Electroacupuncture: 2/15Hz, 2mA	Weight	P<0.05
Han (2014)[51]	SD rats (male)	(10/10)	160 ± 10	High-fat diet (8w)	Not given	15 minutes, once a day, 20 days	Electroacupuncture: continuous waves of 100Hz of frequency	Weight	P<0.01
Cao (2014)[85]	SD rats (male)	(7/7)	60-80	High-fat diet (3m)	ST36, ST44	15 minutes, 6 days for a week, 4 weeks	Electroacupuncture: 2/15Hz of frequency and current density of 1mA	Weight	P<0.05
Tan (2014)[52]	SD rats (male)	(12/12)	90 ± 10	High-fat diet (5w)	ST25, ST40	20 minutes, once a day, 6 days for a week, 4 weeks	Electroacupuncture: continuous waves of 3Hz of frequency and current density of 1.5V	Lee's index	P<0.05
Gao (2013)[86]	SD rats (male)	(10/10)	190-210	High-fat diet (10w)	ST36, SP6	15 minutes, once a day, 2 weeks	Electroacupuncture: disperse-dense waves of 2Hz of frequency	Weight	P<0.01
Yu (2013)[87]	SD rats (male)	(10/10)	Not given	High-fat diet (3m)	ST36, ST25	15 minutes, once a day, 6 days for a week, 39 days	Electroacupuncture: 2/15Hz of frequency and current density of 2mA	Weight	P<0.01
Tang (2013)[88]	C57BL/6 mice (male)	(6/6)	20 ± 2	High-fat diet (16w)	ST36, SP6, ST25, CV12, ST40, CV4	1 minute, every 10 minutes, twice a day, 10 days	Handle acupuncture: even reinforcing-reducing by twirling for 1 minute, every 10 minutes	Weight	P<0.05
He (2013)[63]	SD rats (male)	(7/7)	65-80	High-fat diet (16w)	ST36, PC6	15 minutes, once a day, 4 weeks	Electroacupuncture: continuous waves of 15Hz of frequency and current density of 1.5V	Weight	P<0.05
Lao (2013)[56]	SD rats (male/female)	(10/10)	200-250	High-fat diet (60d)	CV12, ST36, SP6	15 minutes, once a day, 1 month	Electroacupuncture: disperse-dense waves of 5-10Hz of frequency	1.Weight 2.Lee's index	1.P<0.01 2.p<0.01
He (2013)[76]	SD rats (male)	(10/10)	50-70	High-fat diet (12w)	ST2, ST25, ST36, ST44	15 minutes, once a day, 6 days for a week, 4 weeks	Electroacupuncture: 10Hz of frequency and current density of 1.5V, 3mA	Weight	P<0.05
Jiang (2012)[89]	SD rats (male)	(6/6)	50 ± 10	High-fat diet (90d)	ST36, ST44	Once a week, 2 weeks	Catgut implantation	1.Weight 2.Lee's index	1.P<0.001 2.P<0.001
Luo (2012)[90]	SD rats (male)	(10/10)	60-90	High-fat diet (12w)	ST21, ST25, SP15, ST44	10 minutes, once a day, 2 weeks	Electroacupuncture: continuous waves of 10Hz of frequency and current density of 0.5V	1.Mean weight	P<0.05
Liu (1998)[57]	SD rats (male)	(8/8)	50-60	High-fat diet (3m)	ST36, ST44	10 minutes, once a day, 12 days	Electroacupuncture: continuous waves of 10Hz of frequency and current density of 1.5V	1.Weight 2.Lee's index	1.P<0.001 2.P<0.001
Wei (2002)[60]	SD rats (male)	(14/15)	50-60	High-fat diet (3m)	ST36, ST44	10 minutes, once a day, 12 days	Electroacupuncture: continuous waves of 10Hz of frequency and current density of 1.5V	1.Weight 2.Lee's index	1.P<0.01 2.P<0.001
Yuan (2003)[91]	SD rats (male)	(8/8)	62-71	High-fat diet (90d)	ST36, CV12, GB41	15 minutes, once a day, 15 days	Electroacupuncture: continuous waves of 8Hz of frequency and current density of 1V	1.Weight 2.Lee's index	1.P<0.001 2.P<0.001
Li (2003)[92]	SD rats (male)	(10/10)	50 ± 5	High-fat diet (14w)	ST36, SP6	30 minutes, 3 times a week, 4 weeks	Electroacupuncture: 20Hz or 100Hz of frequency and increasing density of 0.5-1-1.5mA, each intensity for 10 minutes	Weight	P<0.05
Tian (2005)[93]	SD rats (male)	(10/10)	40-50	High-fat diet (16w)	ST36, SP6	30 minutes, 3 times a week, 4 weeks	Electroacupuncture: continuous waves of 2Hz of frequency and increasing density of 0.5-1-1.5mA, each intensity for 10 minutes	Weight	P<0.05
Huo (2011)[64]	SD rats (female)	(14/10)	180 ± 20	High-fat diet	ST36, ST25,	Every 10 days, 30 days	Catgut implantation	1.Weight 2.Lee's index	1. p≤0.01 2. p≤0.01
Zhao (2001)[59]	SD rats (male)	(13/13)	50	High-fat diet (3m)	ST36, ST44	20 minutes, once a day, 12 days	Electroacupuncture: continuous waves of 10Hz of frequency and current density of 1V	Weight change	P<0.01
Sun (2005)[65]	SD rats (male)	(12/12)	60-100	High-fat diet	ST36, ST44	10 minutes, once a day, 14 days	Handle acupuncture	1.Weight 2.Lee's index	1.P<0.001 2.P<0.001
Chen (2005)[42]	SD rats (male)	(8/7)	50-70	High-fat diet (45d)	ST36, ST44	20 minutes, twice a day, 15 days	Handle acupuncture: lifting and thrusting for 20 minutes every 5 minutes	1.Weight 2.Lee's index	1.P<0.05 2.P<0.05
Zhang (2006)[66]	SD rats (male)	(8/8)	50-60	High-fat diet (13w)	ST36, ST44	10 minutes, once a day, 2 weeks	Electroacupuncture: continuous waves of 10Hz of frequency and current density of 1.5V	1.Weight 2.Lee's index	1.P<0.01 2.P<0.05
Yang (2006)[53]	Wistar rats (male)	(15/15)	60-100	High-fat diet (30d)	ST36, CV12	10 minutes, once a day, 14 days	Electroacupuncture: 10Hz of frequency and current density of 1.5V	1.Weight 2.Lee's index	1.P<0.01 2.P<0.01
Gao (2006)[48]	SD rats (male)	(10/10)	80 ± 10	High-fat diet (3m)	ST36, ST44, ST25,	10 minutes, once a day, 14 days	Electroacupuncture: continuous waves of 1Hz of frequency and current density of 1.5V	1.Weight 2.Lee's index	1.P<0.01 2.P<0.01
Li (2006)[67]	SD rats (male)	(20/20)	55-76	High-fat diet (3m)	ST36, ST44	10 minutes, once a day, 15 days	Electroacupuncture: continuous waves of 10Hz of frequency and current density of 1.5V	1.Weight 2.Lee's index	1.P<0.01 2.P<0.01
Zhang (2006)[94]	SD rats (male)	(6/6)	60-90	Referring to Liu Zhicheng's methods	ST36, ST44	10 minutes, once a day, 3 weeks	Electroacupuncture: continuous waves of 10Hz of frequency and current density of 1.5V	1.Weight 2.Lee's index	1.P<0.01 2.P<0.01
Zhong (2007)[68]	SD rats (male)	(6/6)	50-70	High-fat diet (3m)	ST36, ST25, SP6	20 minutes, once a day, 10 days	Electroacupuncture: disperse-dense waves and current density of 0.3-0.6mA	1.Weight 2.Lee's index	1.P<0.01 2.P<0.01
Qi (2007)[77]	SD rats (male/female)	(10/10)	170 ± 20	High-fat diet (4w)	ST36, PC6	15 minutes, 14 days	Electroacupuncture: discontinuous waves of 2Hz of frequency and current density of 1.5V, 1mA	Weight	P<0.05
Wu (2008)[43]	Wistar rats (male)	(10/6)	60-100	High-fat diet	CV12, ST25, CV6, LI11, ST36	30 minutes, once a day, 4 times	Handle acupuncture: retaining time of 30 minutes	1.Weight 2.Lee's index	1.P<0.01 2.P<0.01
Li (2008)[49]	SD rats (male)	(8/8)	60 ± 10	Referring to Liu Zhicheng's methods	ST36, SP6	30 minutes, once a day, 28 days	Electroacupuncture: disperse-dense waves of 20Hz of frequency and current density of 2mA	1.Weight 2.Lee's index	1.P<0.01 2.P<0.01
Yang (2008)[95]	Wistar rats (male)	(8/8)	60 ± 5	High-fat diet (30d)	ST25, ST36, ST34	every 10 days, 3 times	Catgut implantation	1.Weight 2.Lee's index	1.P<0.05 2.P<0.05
Yao (2004)[69]	SD rats (male)	(8/8)	70-80	High-fat diet (4m)	ST36, ST44, ST25,	10 minutes, once a day, 12 days	Electroacupuncture + auricular acupuncture: continuous waves of 1Hz of frequency and current density of 1.5V	1.Weight 2.Lee's index	1.P<0.05 2.P<0.01
Gong (2008)[96]	SD rats (male)	(15/15)	50-70	High-fat diet (4m)	ST36, ST44	15 minutes, once a day, 6 days for a week, 39 days	Electroacupuncture: 2/15Hz of frequency and current density of 1.5V, 2mA	Weight	P<0.01
Yang (2007)[97]	SD rats (male)	(10/10)	55 ± 5	High-fat diet (12w)	ST36, ST25	20 minutes, once a day, 15 days	Electroacupuncture: continuous waves of 5Hz of frequency	1.Weight 2.Lee's index	1.P<0.05 2.P<0.05
Gao (2011)[98]	SD rats (male)	(12/12)	180-200	High-fat diet (10w)	ST36, ST25, BL20	Electroacupuncture group: 10 minutes, once a day, 15 days; catgut implantation group: every 7 days, twice	Electroacupuncture: continuous waves of 2Hz of frequency; catgut implantation	Weight	P<0.01
Yu (2011)[99]	SD rats (male)	(10/10)	150-170	High-fat diet (8w)	ST36, SP6	15 minutes, once a day, 14 days	Electroacupuncture: continuous waves of 20Hz of frequency and current density of 5V	1.Weight 2.Lee's index	1.P<0.01 2.P<0.01
Liu (2011)[100]	SD rats (male)	(10/10)	60-100	High-fat diet (30d)	ST36, SP6	10 minutes, once a day, 14 days	Electroacupuncture: continuous waves of 10Hz of frequency and current density of 2V	Lee's index	P<0.01
Xu (2007)[101]	SD rats (male)	(18/18)	Not given	High-fat diet (3m)	ST36, ST44	15 minutes, 6 days for a week, 49 days	Electroacupuncture: 2/15Hz, 4mA	Weight	p≤0.001
Zhan (2000)[61]	SD rats (male)	(8/8)	50-60	High-fat diet (3m)	ST36, ST44	5 minutes, 12days	Electroacupuncture: continuous waves of 10Hz of frequency and current density of 1.5V	Weight	P<0.001
Tian (2005)[102]	SD rats (male)	(20/20)	45-55	High-fat diet (14w)	ST36	30 minutes, three times a week, 4 weeks	Electroacupuncture: 2-100Hz,0.5-1.0-1.5mA	Weight	P<0.001
Zhang (2013)[70]	SD rats (female)	(8/8)	80 ± 10	High-fat diet (35d)	SP6, CV12, ST40	15 minutes, once a day, 10 days	Electroacupuncture: disperse-dense waves of 4-20Hz of frequency and current density of 1-2V	1.Weight 2.Lee's index	1.P<0.01 2.P<0.01
Wang (2008)[103]	SD rats (male)	(12/12)	50-70	High-fat diet (4w)	CV12, CV4, ST36	Electroacupuncture group: 20 minutes, twice a day, 4 weeks; catgut implantation group: every 7 days, 4 times	Catgut implantation	1.Weight 2.Lee's index	1.P<0.01 2.P<0.01
Yu (2008)[104]	SD rats (male)	(8/8)	60 ± 10	High-fat diet (6w)	ST36, ST44, ST40, SP9, CV4, CV12	10 minutes, once a day, 10 days	Electroacupuncture: 2/100Hz of frequency and current density of 2mA	1.Weight growth	P<0.05
Chen (2012)[58]	SD rats (male/female)	(10/10)	200-250	High-fat diet (2m)	CV12, ST36, SP6	20 minutes, once a day, 1 month	Electroacupuncture: disperse-dense waves of 5-10Hz of frequency	1.Weight 2.Lee's index	1.P<0.05 2.P<0.05
Chen (2012)[105]	SD rats (male)	(8/8)	60 ± 10	High-fat diet (9w)	CV4	15 minutes, once a day, 20 days	Handle acupuncture: retaining time of 15 minutes	Weight	P<0.05
Xu (2005)[71]	SD rats (male)	(8/8)	50	High-fat diet (3m)	ST36, ST44	10 minutes, once a day, 14 days	Electroacupuncture: continuous waves of 10Hz of frequency and current density of 1.5V	1.Weight 2.Lee's index	1.P<0.001 2.P<0.001
Zhang (2012)[106]	Wistar rats (male)	(8/8)	50 ± 5	High-fat diet (10w)	ST36	20 minutes, once a day, 30 days	Electroacupuncture: continuous waves of 10Hz of frequency	1.Weight 2.Lee's index	1.P<0.05 2.P<0.05
Luo (2013)[107]	SD rats (male/female)	(22/22)	80-120	High-fat diet (4m)	ST36, ST44, ST25,	30 minutes, once a day, 12 days	Handle acupuncture: ST36,ST 25,even reinforcing-reducing by twirling for 1 minute; ST44 reducing by twirling for 0.5 minute; retaining time of 30 minutes	Weight	P<0.000l
Liu (2003)[62]	SD rats (male)	(12/12)	50-70	High-fat diet (3m)	ST36, ST44	10 minutes, once a day, 14 days	Electroacupuncture: continuous waves of 10Hz of frequency and current density of 1.5V	1.Weight 2.Lee's index	1.P<0.01 2.P<0.01
Tao (2013)[108]	SD rats (male/female)	(15/15)	50 ± 5	High-fat diet (14w)	ST36, GB41, CV12, CV4	15 minutes, once a day, 20 days	Electroacupuncture: continuous waves of 10Hz of frequency and current density of 1.5V	1.Weight 2.Lee's index	1.P>0.05 2.P>0.05
Lin (2012)[109]	SD rats (male)	(6/8)	60-70	High-fat diet (12w)	Not given	Once a day, 30 times	Electroacupuncture: disperse-dense waves of 2/15Hz of frequency and current density of 3mA	Weight	P<0.05
Shao (2008)[78]	SD rats (male)	(15/15)	70—90	High-fat diet (16w)	Stomach area and Hunger Point	15 minutes, once a day, 6 days for a week, 7 weeks	Electroacupuncture: disperse-dense waves of 2/15Hz of frequency and current density of 3mA	Weight	P<0.05
Huang (2007)[79]	SD rats (male/female)	(15/20)	60 ± 5	High-fat diet (12w)	SP6, ST25	30 minutes, once a day, 15 days	Electroacupuncture: disperse-dense waves of 2-20Hz of frequency and current density of 2-3mA	1.Weight 2.Lee's index	1.P<0.05 2.P<0.05
Wang (2009)[54]	SD rats (male)	(12/12)	50-70	High-fat diet (4w)	ST36, CV12, CV4	20 minutes, twice a day, 4 weeks	Electroacupuncture: continuous waves of 10Hz of frequency and current density of 1.5-2.5V	1.Weight 2.Lee's index	1.P<0.01 2.P<0.05
Hong (2003)[110]	SD rats (male)	(10/10)	60-90	High-fat diet (10w)	ST44, ST36, CV4	10 minutes, once a day, 15 days	Electroacupuncture: continuous waves of 10Hz of frequency and current density of 1.5V	1.Weight 2.Lee's index	1.P<0.001 2.P<0.01
Zhang (2006)[111]	SD rats (male)	(8/8)	50 ± 5	High-fat diet (4w)	ST36, CV12, CV9, CV6	10 minutes, once a day, 4 weeks	Handle acupuncture: even reinforcing-reducing by twirling for 1 minute, every 10 minutes	1.Weight 2.Lee's index	1.P<0.05 2.P<0.05
Fu (2005)[112]	SD rats (male)	(12/11)	69-96	High-fat diet (12w)	ST36, ST44	20 minutes, 15 days	Electroacupuncture: continuous waves of 10Hz of frequency and current density of 1.5V	1.Weight 2.Lee's index	1.P<0.01 2.P<0.01
TAKEMASA SHIRAISHI (1995)[113]	Wistar rats (male)	(21/10)	80-90	High-fat food (31w)	Not given	Not given	Pulse duration 0.01-5.0 ms; voltage 5-40 V: frequency, single pulse or 0.5-50 Hz	Weight	P<0.01
Martin S. Mok (1976)[44]	Guinea pigs	(3/3)	Not given	Feeding regimen (2m)	Not given	3 mins every day, for 3 weeks	Electroacupuncture: 100Hz	Weight	P>0.05
Yan (2013)[114]	SD rats (male)	(12/12)	52 ± 9	High-fat diet (12w)	ST36, ST44	Once a week, 4 weeks	Catgut implantation	1.Weight 2.Lee's index	1.P<0.01 2.P<0.01
Bo Ji (2013)[115]	Rats	(5/6)	180-200	High fat diet (1w)	Not given	20 min and conducted twice, separated by an 80 min rest interval every time, lasting for 7 days	Electroacupuncture	Weight	P<0.05
Shen (2014)[116]	C57BL/6J mice	(28/28)	Not given	Fed D12451 rodent diet with 45 kcal% fat for eight weeks	Not given	30 minutes, once a day, six days a week, for 4 weeks	Electroacupuncture: 2/15Hz, 1mA	Weight ration	P<0.05
Wu (2009)[117]	SD rats (male/female)	(15/15)	50 ± 5	High-fat diet (30d)	ST36, CV12	Once a week, 4 weeks	Catgut implantation	1.Weight 2.Lee's index	1.P<0.01 2.P<0.01
Alfredo Eduardo Orozco Terån (2003)[118]	Wistar rats (female)	(8/8)	50-60	High-fat diet (12w)	ST36, ST44, SP6	10 minutes, once a day, 10 days	Electroacupuncture: 10Hz, 1.5V	1.Weight 2.Lee's index	1.P<0.001 2.P<0.001
Liu (2016)[80]	SD rats (male)	(10/10)	100	High-fat diet	ST36, LI11	20 minutes, once a day, 30 days	Electroacupuncture: 10Hz, 1.5mA	Weight	P<0.05
Si (2016)[72]	C57BL/6 mice (male/female)	(6/6)	18 ± 2	High-fat diet (8w)	ST25, CV4, ST36, SP6	10 minutes, once a day, 7 days	Electroacupuncture: disperse-dense waves of 30Hz of frequency and current density of 2-3mA	1.Weight 2.Lee's index	1.P<0.05 2.P<0.05
Leng (2015)[119]	SD rats (male/female)	(7/7)	50-80	High-fat diet (12w)	ST25, SP6, ST36, CV12	Once a day, 5 days for a session, 4 sessions	Electroacupuncture: 18Hz, 2mA	Weight	P<0.05
Gong (2011)[81]	SD rats (male)	(10/10)	50-70	High-fat diet (4m)	ST36, ST44, CV12	15 minutes, once a day, 6 days for a week, 39 days	Electroacupuncture: 2mA	1.Weight 2.Lee's index	1.P<0.01 2.P<0.01
Deng (2016)[120]	C57BL/6J mice (male)	(6/6)	9	High-fat diet (10w)	ST36	Every 10 days, 3 times	Catgut implantation	Weight	P<0.01
Sun (2013)[121]	SD rats (male)	(5/5)	80-90	High-fat diet (5w)	ST36, SP6	30 minutes, three times a day, 6 weeks	Electroacupuncture: 10Hz, 0.6ms, 0.5-1.0-1.5mA	Weight	P<0.05
Li (2012)[122]	SD rats (male)	(15/15)	70 ± 10	High-fat diet (13w)	ST36, SP6, ST25, CV12, ST40, CV4	10 minutes, twice a day, 9 weeks	Handle acupuncture: even reinforcing-reducing by twirling for 1 minute, every 10 minutes	1.Weight 2.Lee's index	1.P<0.01 2.P<0.01
Tan (2008)[55]	SD rats (male)	(8/8)	50 ± 5	High-fat diet (14w)	SP6, ST36	30 minutes, once a day, 4 weeks	Electroacupuncture: disperse-dense waves of 20Hz of frequency and current density of rise of 1mA every 10 minutes from 2mA	1.Weight 2.Lee's index	1.P<0.01 2.P<0.01
Yao (2005)[123]	SD rats (male)	(8/8)	70-80	High-fat diet (4m)	ST36, ST44, ST25	10 minutes, once a day, 12 days	Electroacupuncture: continuous waves of 1Hz of frequency and current density of 1.5V	1.Weight 2.Lee's index	1.P<0.01 2.P<0.01
Yuan (2005)[45]	SD rats (male)	(10/16)	Not given	High-fat diet (3m)	ST36, ST44	15 minutes, once a day, 6 days for a week, 7 weeks	Electroacupuncture	Weight change	P<0.01
Su (1999)[124]	SD rats (male)	(14/16)	50-60	Referring to Liu Zhicheng's methods	ST36, ST44	5 minutes, once a day, 12days	Electroacupuncture: continuous waves of 10Hz of frequency and current density of 1V	1.Weight 2.Lee's index	1.P<0.001 2.P<0.001
Yin (2008)[46]	SD rats (male)	(6/5)	50-70	High-fat diet (7w)	ST36, CV12	10 minutes, once a day, 15 days	Electroacupuncture: 5Hz	1.Weight 2.Lee's index	1.P<0.05 2.P<0.05
Liu (2007)[125]	SD rats (male)	(8/8)	180 ± 20	High-fat diet (8w)	ST36, ST40, CV12, ST29	Electroacupuncture group:15 minutes, once a day, 3 weeks for a session, 3 sessions; catgut implantation group: every 15 days, 3 times	Catgut implantation	1.Weight 2.Lee's index	1.P<0.05 2.P<0.05
Fang (2005)[126]	SD rats (male/female)	(10/10)	180 ± 40	High-fat diet (8w)	ST36, ST44	30 minutes, once a day, 4 weeks	Electroacupuncture: frequency mixing of 2-100Hz	1.Weight 2.Lee's index	1.P<0.01 2.P<0.01
Pan (2015)[73]	SD rats	(10/10)	180	High-fat diet (8w)	ST25, CV12, SP9	Every 10days, 3times	Catgut implantation	Weight	P<0.05
Han Li M.M (2015)[82]	SD rats (male)	(12/12)	100-120	High-fat diet (5w)	Not given	30 min of electrical stimulation daily for 6 wk	Electroacupuncture: 2/15Hz, 2mA	Weight	P<0.001
Wen Chorng-Kai (2014)[127]	SD rats (male)	(5/5)	130 ± 12	High-fat diet (15w)	Not given	20 min, once per day for 3 or 7 consecutive days	Electroacupuncture: 10Hz, 0.5-1.0mA	Weight	P<0.05
Si (2014)[74]	SD rats (male)	(10/10)	160 ± 10	High-fat diet (10w)	CV12, ST25, CV4, SP15, ST36, SP6, CV12, CV4	15 minutes, once a day, 20 days	Electroacupuncture: continuous waves of 100Hz of frequency	Weight	P<0.01
Wang (2009)[128]	Wistar rats (male)	(8/8)	50 ± 5	High-fat diet (10w)	The acupoints of Spleen, Stomach, and Large Intestine Channel	20 minutes, once a day, 30 days	Handle acupuncture	1.Weight 2.Lee's index	1.P<0.001 2.P<0.001
Ge (2007)[129]	SD rats	(19/19)	Not given	Injecting 15% monosodium glutamate, 0.2mL/10g (5d)	ST36, SP6, CV4, CV12	10 minutes, once a day, 28 days	Electroacupuncture: disperse-dense waves of 100Hz of frequency	Lee's index	P<0.05
Wang (2005)[50]	SD rats	(20/20)	Not Given	Injecting 15% monosodium glutamate, 0.3mL/10g (5d)	ST36, SP6, CV4, CV12	16 minutes, once a day, 28 days	Electroacupuncture	1.Weight 2.Lee's index	1.P<0.01 2.P<0.01
Zheng (2015)[130]	C57BL/KsJ ob/ob mice	(6/6)	Not given	Not given	ST36, SP6, CV12, CV4	10 minutes, once a day, 8 weeks	Electroacupuncture: continuous waves of 10-30Hz of frequency	1.Weight 2.Lee's index	1.P<0.05 2.P<0.05
Zheng (2006)[131]	SD rats (male)	(8/8)	551.968 ± 47.196	Not given	ST36, ST25, CV12, SP9	10 minutes, twice a day, 12 weeks	Electroacupuncture: 10Hz, 1.5V	1.Weight 2.Lee's index	1.P<0.001 2.P<0.01
Sun (2011)[75]	SD rats (male)	(10/10)	180	High-fat diet (6w)	ST25, ST36	10 minutes, once a day in the first 10 days, twice a day in the next 20 days	Handle acupuncture	1.Weight 2.Lee's index	1.P<0.05 2.P<0.05
Wang (2018)[132]	Wistar rats (male)	(8/8)	180-220	High-fat diet (12w)	ST25, ST36	30 minutes, 5 days a week, 8 weeks	Electroacupuncture: frequency of 2 Hz/15 Hz and intensity of 1 m A	Weight	P<0.05
Si (2018)[133]	C57BL/6 mice (male/female)	(10/10)	18 ± 2	High-fat diet (8w)	ST25, CV4, ST36, SP6	10 minutes, once a day, 21 days	Electroacupuncture: disperse-dense waves, current density of 2-3mA	1.Weight 2.Lee's index	1.P<0.01 2.P<0.01
Tang (2017) [134]	C57BL/6 mice (male)	(6/12)	10.2 ± 0.68	High-fat diet (12w)	ST36, ST44	20 minutes, 6 days a week, 4 weeks	Electroacupuncture: 2Hz/15 Hz HzH of frequency, current density of 0.6-1mA	Weight	P<0.01
Wu (2019) [135]	Rats (male)	(8/8)	110-120	High-fat diet (12w)	ST40	20 minutes, three times a week, 12 weeks	Handle acupuncture	Weight	P<0.05
Si (2019)[136]	C57BL/6 mice (male/female)	(10/10)	18 ± 2	High-fat diet (8w)	CV12, CV4, ST25, ST36,	10 minutes, once a day, 28 days	Handle acupuncture	1.Weight 2.Lee's index	1.P<0.01 2.P<0.01
Yao (2019)[137]	SD rats (male)	(8/8)	70-90	High-fat diet (14w)	ST25, CV12, ST36, SP6	20 minutes, once a day, 28 days	Electroacupuncture: 18 Hz HzH of frequency, current density of 2mA	Weight	P<0.05
Zhang (2017)[138]	C57BL/6 mice (male)	(10/10)	22 ± 2	High-fat diet (16w)	CV9, ST36, EX-B3	15 minutes, once a day, 8 weeks	Handle acupuncture	Weight	P<0.05
Yang (2018)[139]	Wistar rats (male)	(10/10)	Not given	High-fat diet (8w)	ST44, CV12, CV4, ST36	10 minutes, three times a week, 8 weeks	Electroacupuncture: 2 Hz HzH of frequency, current density of 1mA	1.Weight 2.Lee's index	1.P<0.05 2.P<0.05
Li (2017) [140]	SD rats (male)	(8/8)	Not given	High-fat diet (5w)	CV12, ST25	Once a week, 4 weeks	Catgut implantation	1.Weight 2.Lee's index	1.P<0.01 2.P<0.01
Fan (2018) [141]	SD rats (male)	(7/7)	120~150 (±30 )	High-fat diet (7w)	CV12, ST25, CV4, ST36, SP9, ST40	30 minutes, once a day, 4 weeks	Handle acupuncture	1.Weight 2.Lee's index	1.P<0.01 2.P<0.01
Lin (2018) [142]	SD rats (male)	(10/10)	120-150	High-fat diet (7w)	CV12, ST25, CV4, ST36, SP9, ST40	30 minutes, once a day, 28 days	Handle acupuncture	Weight	P<0.01
He (2018) [143]	SD rats (male)	(10/10)	120~150 (±30 )	High-fat diet (7w)	CV12, ST25, CV4, ST36, SP9, ST40	30 minutes, once a day, 28 days	Handle acupuncture	Weight	P<0.01
Li (2016) [144]	SD rats (male)	(10/9)	Not given	High-fat diet (12w)	CV12, ST25, ST36, SP6	20 minutes, once a day, 4 weeks	Handle acupuncture	Weight	P>0.05
Zhou (2016) [145]	Wistar rats (male)	(10/10)	160 ± 20	High-fat diet (8w)	CV4, CV12, ST36, ST40	10 minutes, three times a week, 4 weeks	Electroacupuncture: 2 Hz HzH of frequency, current density of 1mA	Weight	P<0.01
Wei (2017) [146]	SD rats (male)	(6/6)	50-60	High-fat diet (12w)	ST36, ST40	30 minutes, once a day, 4 weeks	Electroacupuncture: 2 /15Hz HzH of frequency, current density of 2mA	Weight	P<0.05
Wang (2016) [147]	SD rats (male)	(11/11)	190.41 ± 2.23	High-fat diet (10w)	CV12, CV6, ST25	30 minutes, once a day, 2 weeks	Electroacupuncture: current density of 1mA	1.Weight 2.Lee's index	1.P<0.05 2.P<0.05
Xu (2017) [148]	C57BL/6 mice (male)	(8/8)	9	High-fat diet (10w)	ST36, ST44	15 minutes, once a day, 4 weeks	Electroacupuncture: 2 /15Hz of frequency, current density of 1mA	Weight	P<0.01

**Table 2:** Quality assessment of included studies.

Study	(1)	(2)	(3)	(4)	(5)	(6)	(7)	(8)	(9)	(10)	Total
Tang (2013)[88]	YES	YES	YES	YES	NO	NO	YES	YES	YES	YES	8
Yan (2015)[27]	YES	NO	YES	YES	NO	NO	YES	YES	YES	YES	7
Yin (2015)[84]	YES	NO	YES	YES	NO	NO	YES	YES	YES	YES	7
Ran (2014)[47]	YES	YES	YES	YES	NO	NO	NO	YES	YES	YES	7
Gao (2013)[86]	YES	YES	YES	YES	NO	NO	NO	YES	YES	YES	7
He (2013)[76]	YES	YES	YES	YES	NO	NO	NO	YES	YES	YES	7
Jiang (2012)[89]	YES	YES	YES	YES	NO	NO	NO	YES	YES	YES	7
Luo (2012)[90]	YES	YES	YES	YES	NO	NO	NO	YES	YES	YES	7
Gong (2008)[96]	YES	NO	YES	YES	NO	NO	YES	YES	YES	YES	7
Yu (2011)[99]	YES	NO	YES	YES	NO	NO	YES	YES	YES	YES	7
Tian (2005)[102]	YES	YES	YES	YES	NO	NO	NO	YES	YES	YES	7
Wang (2009)[54]	YES	YES	YES	YES	NO	NO	NO	YES	YES	YES	7
Liu (2016)[80]	YES	YES	YES	YES	NO	NO	NO	YES	YES	YES	7
Si (2016)[72]	YES	YES	YES	YES	NO	NO	NO	YES	YES	YES	7
Leng (2015)[119]	YES	YES	YES	YES	NO	NO	NO	YES	YES	YES	7
Sun (2013)[121]	YES	YES	YES	YES	NO	NO	NO	YES	YES	YES	7
Han Li M.M (2015)[82]	YES	YES	YES	YES	NO	NO	YES	NO	YES	YES	7
Han (2014)[51]	YES	NO	YES	YES	NO	NO	NO	YES	YES	YES	6
Yu (2013)[87]	YES	NO	YES	YES	NO	NO	NO	YES	YES	YES	6
He (2013)[63]	YES	YES	YES	YES	NO	NO	NO	NO	YES	YES	6
Lao (2013)[56]	YES	NO	YES	YES	NO	NO	NO	YES	YES	YES	6
Liu (1998)[57]	YES	NO	YES	YES	NO	NO	NO	YES	YES	YES	6
Wei (2002)[60]	YES	NO	NO	YES	NO	NO	NO	YES	YES	YES	6
Yuan (2003)[91]	YES	YES	YES	YES	NO	NO	NO	NO	YES	YES	6
Zhao (2001)[59]	YES	NO	YES	YES	NO	NO	NO	YES	YES	YES	6
Chen (2005)[65]	YES	NO	YES	YES	NO	NO	NO	YES	YES	YES	6
Gao (2006)[48]	YES	NO	YES	YES	NO	NO	NO	YES	YES	YES	6
Li (2006)[67]	YES	NO	YES	YES	NO	NO	NO	YES	YES	YES	6
Zhong (2007)[68]	YES	YES	YES	YES	NO	NO	NO	NO	YES	YES	6
Gao (2011)[98]	YES	NO	YES	YES	NO	NO	NO	YES	YES	YES	6
Liu (2011)[100]	YES	NO	YES	YES	NO	NO	NO	YES	YES	YES	6
Chen (2012)[105]	NO	YES	YES	YES	NO	NO	YES	NO	YES	YES	6
Liu (2003)[62]	YES	NO	YES	YES	NO	NO	NO	YES	YES	YES	6
TAKEMASA SHIRAISHI (1995)[113]	YES	YES	YES	YES	NO	NO	YES	NO	YES	NO	6
Gong (2011)[81]	YES	NO	YES	YES	NO	NO	NO	YES	YES	YES	6
Deng (2016)[120]	YES	NO	YES	YES	NO	NO	NO	YES	YES	YES	6
Li (2012)[122]	YES	NO	YES	YES	NO	NO	NO	YES	YES	YES	6
Yuan (2005)[45]	YES	NO	YES	YES	NO	NO	NO	YES	YES	YES	6
Yin (2008)[46]	NO	YES	YES	YES	NO	NO	NO	NO	YES	YES	6
Fang (2005)[126]	YES	NO	YES	YES	NO	NO	NO	YES	YES	YES	6
Chorng-Kai Wen (2014)[127]	YES	NO	YES	YES	NO	NO	YES	NO	YES	YES	6
Ge (2007)[129]	YES	YES	YES	YES	NO	NO	NO	YES	NO	YES	6
Wang (2005)[50]	YES	NO	YES	YES	NO	NO	NO	YES	YES	YES	6
Zheng (2006)[131]	YES	YES	YES	YES	NO	NO	NO	YES	NO	YES	6
Yin (2015)[83]	YES	NO	YES	YES	NO	NO	NO	YES	NO	YES	5
Cao (2014)[85]	NO	YES	YES	YES	NO	NO	NO	NO	YES	YES	5
Tan (2014)[52]	NO	YES	YES	YES	NO	NO	NO	NO	YES	YES	5
Li (2003)[92]	NO	YES	YES	YES	NO	NO	NO	NO	YES	YES	5
Tian (2005)[93]	NO	YES	YES	YES	NO	NO	NO	NO	YES	YES	5
Sun (2005)[65]	YES	NO	YES	YES	NO	NO	NO	YES	NO	YES	5
Zhang (2006)[66]	YES	NO	YES	YES	NO	NO	NO	NO	YES	YES	5
Yang (2006)[53]	YES	NO	YES	YES	NO	NO	NO	NO	YES	YES	5
Zhang (2006)[94]	YES	NO	YES	YES	NO	NO	NO	YES	NO	YES	5
Qi (2007)[77]	YES	NO	YES	YES	NO	NO	NO	NO	YES	YES	5
Li (2008)[49]	YES	NO	YES	YES	NO	NO	NO	YES	NO	YES	5
Yang (2008)[95]	YES	NO	YES	YES	NO	NO	NO	NO	YES	YES	5
Yang (2007)[97]	YES	NO	YES	YES	NO	NO	NO	NO	YES	YES	5
Xu (2007)[101]	YES	NO	YES	YES	NO	NO	NO	NO	YES	YES	5
Zhan (2000)[61]	YES	NO	YES	YES	NO	NO	NO	NO	YES	YES	5
Zhang (2013)[70]	NO	YES	YES	YES	NO	NO	NO	NO	YES	YES	5
Wang (2008)[103]	NO	YES	YES	YES	NO	NO	NO	NO	YES	YES	5
Yu (2008)[104]	NO	YES	YES	YES	NO	NO	NO	NO	YES	YES	5
Chen (2012)[58]	NO	YES	YES	YES	NO	NO	NO	NO	YES	YES	5
Tao (2013)[108]	NO	YES	YES	YES	NO	NO	NO	NO	YES	YES	5
Huang (2007)[79]	YES	NO	NO	YES	NO	NO	NO	YES	YES	YES	5
Zhang (2006)[111]	NO	YES	YES	YES	NO	NO	NO	NO	YES	YES	5
Fu (2005)[112]	YES	NO	YES	YES	NO	NO	NO	NO	YES	YES	5
Martin S. Mok (1976)[44]	YES	NO	YES	YES	NO	NO	NO	NO	YES	YES	5
Yan (2013)[114]	NO	YES	YES	YES	NO	NO	NO	NO	YES	YES	5
Bo Ji (2013)[115]	YES	YES	YES	YES	NO	NO	NO	NO	YES	NO	5
Wu (2009)[117]	NO	YES	YES	YES	NO	NO	NO	NO	YES	YES	5
Alfredo Eduardo Orozco Terån (2003)[118]	NO	YES	YES	YES	NO	NO	NO	NO	YES	YES	5
Tan (2008)[55]	NO	YES	YES	NO	NO	NO	NO	NO	YES	YES	5
Yao (2005)[123]	YES	NO	YES	YES	NO	NO	NO	NO	YES	YES	5
Su (1999)[124]	YES	NO	YES	YES	NO	NO	NO	YES	NO	YES	5
Pan (2015)[73]	YES	NO	YES	YES	NO	NO	NO	NO	YES	YES	5
Si (2014)[74]	NO	YES	YES	YES	NO	NO	NO	NO	YES	YES	5
Sun (2011)[75]	NO	YES	YES	YES	NO	NO	NO	NO	YES	YES	5
Huo (2011)[64]	NO	NO	YES	YES	NO	NO	NO	NO	YES	YES	4
Wu (2008)[43]	YES	NO	YES	YES	NO	NO	NO	NO	NO	YES	4
Yao (2004)[69]	NO	NO	YES	YES	NO	NO	NO	NO	YES	YES	4
Xu (2005)[71]	NO	NO	YES	YES	NO	NO	NO	NO	YES	YES	4
Zhang (2012)[106]	NO	NO	YES	YES	NO	NO	NO	NO	YES	YES	4
Lin (2012)[109]	NO	NO	YES	YES	NO	NO	NO	NO	YES	YES	4
Shao (2008)[78]	NO	NO	YES	YES	NO	NO	NO	NO	YES	YES	4
Hong (2003)[110]	NO	NO	YES	YES	NO	NO	NO	NO	YES	YES	4
Shen (2014)[116]	YES	NO	YES	YES	NO	NO	NO	NO	YES	NO	4
Liu (2007)[125]	YES	NO	YES	YES	NO	NO	NO	NO	YES	NO	4
Wang (2009)[128]	NO	NO	YES	YES	NO	NO	NO	NO	YES	YES	4
Zheng (2015)[130]	YES	NO	YES	YES	NO	NO	NO	NO	NO	YES	4
Luo (2013)[107]	NO	NO	NO	YES	NO	NO	NO	NO	YES	YES	3
Wang (2018)[132]	YES	YES	YES	YES	NO	NO	NO	NO	YES	YES	6
Si (2018)[133]	YES	NO	YES	YES	NO	NO	NO	NO	YES	YES	5
Tang (2017)[134]	YES	YES	YES	YES	NO	NO	NO	NO	YES	NO	5
Wu (2019) [135]	YES	YES	YES	YES	NO	NO	YES	YES	YES	YES	8
Si (2019)[136]	YES	YES	YES	YES	NO	NO	NO	NO	YES	YES	6
Yao (2019)[137]	YES	YES	YES	YES	NO	NO	NO	NO	YES	YES	6
Zhang (2017)[138]	YES	YES	YES	YES	NO	NO	NO	NO	YES	YES	6
Yang (2018)[139]	YES	YES	YES	YES	NO	NO	NO	NO	YES	NO	5
Li (2017)[140]	YES	NO	YES	YES	NO	NO	YES	NO	YES	YES	6
Fan (2018) [141]	NO	YES	YES	YES	NO	NO	NO	NO	YES	YES	5
Lin (2018) [142]	NO	YES	YES	YES	NO	NO	NO	NO	YES	YES	5
He (2018) [143]	NO	YES	YES	YES	NO	NO	NO	NO	YES	YES	5
Li (2016) [144]	YES	YES	YES	YES	NO	NO	NO	NO	YES	YES	6
Zhou (2016) [145]	YES	NO	YES	YES	NO	NO	NO	NO	YES	YES	5
Wei (2017) [146]	NO	YES	YES	YES	NO	NO	YES	NO	YES	YES	6
Wang (2016) [147]	YES	NO	YES	YES	NO	NO	NO	NO	YES	YES	5
Xu (2017) [148]	YES	NO	YES	YES	NO	NO	NO	NO	YES	YES	5

Notes: studies fulfilling the criteria of the following:(1) peer-reviewed publication,(2) control of temperature,(3) exclusion of stress reaction, (4) random allocation to treatment or control,(5) blind method,(6) sample size calculation,(7) compliance with animal welfare regulations,(8) statement of research support,(9) detailed modeling method, and (10) complete testing data.

## Data Availability

The datasets supporting the conclusions of this article are included within the article.

## Abbreviations

RCTs: Randomized controlled trials
CNKI: China National Knowledge Infrastructure
VIP: Chinese Science and Technology Periodical Database
CBM: Chinese Biology Medicine Disc
RR: Relative risk
SMD: Standard mean difference
CI: Confidence interval
BMI: Body mass index
SD: Sprague Dawley
TCM: Traditional Chinese Medicine
NPY: Neuropeptide Y
LP: Leptin
EGG: Electrogastrogram
LHA: Lateral hypothalamic area
VNH: Ventromedial hypothalamus
5-HT: 5-Hydroxytryptamine
CCK: Cholecystokinin
VIP: Vasoactive intestinal peptide
IR: Insulin resistance.
